# Biology-guided radiotherapy in metastatic prostate cancer: time to push the envelope?

**DOI:** 10.3389/fonc.2024.1455428

**Published:** 2024-09-09

**Authors:** Andrea Lancia, Gianluca Ingrosso, Beatrice Detti, Eleonora Festa, Elisabetta Bonzano, Flavia Linguanti, Federico Camilli, Niccolò Bertini, Salvatore La Mattina, Carolina Orsatti, Giulio Francolini, Elisabetta Maria Abenavoli, Lorenzo Livi, Cynthia Aristei, Dorine de Jong, Karine A. Al Feghali, Shankar Siva, Carlotta Becherini

**Affiliations:** ^1^ Department of Radiation Oncology, San Matteo Hospital Foundation Istituto di Ricovero e Cura a Carattere Scientifico (IRCCS), Pavia, Italy; ^2^ Radiation Oncology Section, University of Perugia, Perugia, Italy; ^3^ Radiotherapy Unit Prato, Usl Centro Toscana, Presidio Villa Fiorita, Prato, Italy; ^4^ Nuclear Medicine Department, Ospedale San Donato, Arezzo, Italy; ^5^ Radiation Oncology Unit, Oncology Department, Azienda Ospedaliero Universitaria Careggi, Florence, Italy; ^6^ Nuclear Medicine Division, Careggi University Hospital, Florence, Italy; ^7^ Medical Affairs, RefleXion Medical, Inc., Hayward, CA, United States; ^8^ Department of Radiation Oncology, Sir Peter MacCallum Cancer Centre, Melbourne, VIC, Australia; ^9^ Sir Peter MacCallum Department of Oncology, University of Melbourne, Melbourne, VIC, Australia

**Keywords:** radiotherapy, prostate cancer, PET/CT, biology-guided radiotherapy, metastases

## Abstract

The therapeutic landscape of metastatic prostate cancer has undergone a profound revolution in recent years. In addition to the introduction of novel molecules in the clinics, the field has witnessed a tremendous development of functional imaging modalities adding new biological insights which can ultimately inform tailored treatment strategies, including local therapies. The evolution and rise of Stereotactic Body Radiotherapy (SBRT) have been particularly notable in patients with oligometastatic disease, where it has been demonstrated to be a safe and effective treatment strategy yielding favorable results in terms of disease control and improved oncological outcomes. The possibility of debulking all sites of disease, matched with the ambition of potentially extending this treatment paradigm to polymetastatic patients in the not-too-distant future, makes Biology-guided Radiotherapy (BgRT) an attractive paradigm which can be used in conjunction with systemic therapy in the management of patients with metastatic prostate cancer.

## Graphical Abstract

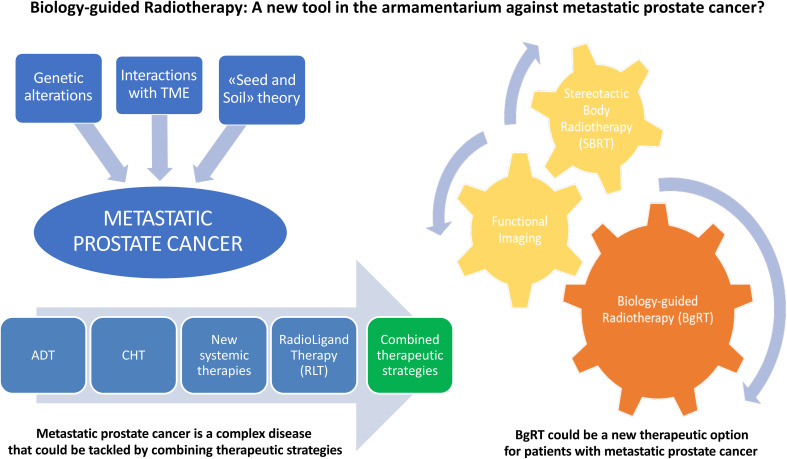

## Introduction: metastatic prostate cancer: definition, biology and concept of oligometastatic state

Prostate cancer (PCa) is the most common type of cancer in men, with more than 280,000 new cases expected to be diagnosed in the United States in 2023, according to the American Cancer Society (1). While the majority of patients with localized disease have an excellent prognosis and may not even require active treatment, some forms are more aggressive and necessitate prompt intervention to prevent metastasis formation.

Metastatic prostate cancer refers to cancer that has spread from the prostate to other parts of the body, such as bones, lymph nodes, or visceral organs (2). This colonization of neoplastic cells represents a serious and often life-threatening condition, despite the currently wider therapeutic armamentarium that physicians have at their disposal.

The biology of metastatic prostate cancer is complex and multifaceted. It is believed that cancer cells can spread from the prostate via lymphatic system and blood vessels. According to the “Seed and Soil” theory, once these cells reach a favorable area, they interact and cooperate with the host micro-environment to form a new tumor deposit (3). At this level, the cross talk between resident immune cells and the tumor is key in determining further spreading of the disease to other organs and tissues.

Different somatic and germline mutations may lead to androgen receptor overexpression resulting in prostate cells overgrowth and tumorigenesis promotion, even in the absence of testosterone (4). Besides, the loss of suppressor genes such as PTEN and TP53 may also be observed in metastatic prostate cancer, as these genes normally play key roles in regulating cells’ growth and division (5).

Understanding genetic alterations, along with a careful evaluation of the interplay between the tumor and its microenvironment is therefore of paramount importance to elucidate the mechanisms behind metastasis. This knowledge could lead to the development of new and more effective systemic treatments for patients with stage IV prostate cancer, who are generally offered palliative therapy.

Nevertheless, the existence of an intermediate stage of disease has been hypothesized during recent years; the concept of the so-called “oligometastatic state” has emerged to identify those patients presenting with a limited number of metastases (usually up to three or five lesions) and who may be amenable to local ablative therapy (6), such as surgery or Stereotactic Body Radiotherapy (SBRT). The latter represents a radiotherapeutic approach that can accurately target and destroy the metastatic foci with curative doses aiming at potentially prolonging survival, at least in a subset of these patients, and improving their quality of life (7–9).

While there is a growing body of evidence supporting the integration of SBRT in treatment strategies for oligometastatic prostate cancer, the decision to pursue focal ablative treatment in this patient group should be made on a case-by-case basis. Factors to consider include location and size of the metastases, natural history of the disease and the possibility to integrate systemic therapy, together with a thorough evaluation of patients’ overall performance status and life expectancy (10).

Biology-guided radiotherapy (BgRT) represents an innovative approach in radiation oncology that aims to personalize and optimize cancer treatment based on the biology of each patient’s tumor. Radiotherapy traditionally consists of the delivery of a defined dose of radiation to the tumor, taking into account its size and anatomical location. In the setting of metastatic cancer, the logistical limitations of present-day technology have restricted its use to the palliation of symptomatic metastases, in which simple techniques and low doses are generally required. Therefore, there is a wide range of patients with stage IV cancer who are currently not deemed eligible for radiotherapy. One of the key components of BgRT is the use of advanced imaging techniques such as positron emission tomography (PET), which provides detailed information about the tumor’s metabolic activity, oxygen levels, and other biological features, depending on the type of radiotracer used. Moreover, by transforming tumors into their own fiducials after intravenous injection of a radiotracer, BgRT has the potential to simplify the process of radiotherapy delivery to multiple sites of disease throughout the body in the same treatment session and to track tumors in real time. The integration of imaging and biological data in BgRT enables clinicians to create treatment plans that are tailored to each patient’s tumor (11). This approach offers several potential benefits. Mainly, it could allow for more accurate targeting of the tumor, minimizing side effects due to radiation exposure of the surrounding healthy tissues. This precision can lead to a tangible improvement in patients’ quality of life during and after treatment. Nonetheless, the rationale behind BgRT could be crucial in the management of patients affected by metastatic disease and could make us rethink the role of radiotherapy in this clinical scenario. In recent years, several studies demonstrated the safety and efficacy of Stereotactic Body Radiotherapy (SBRT) as a metastasis-directed therapy (MDT) delivered with ablative intent in patients affected by metastatic cancer and more specifically in the oligometastatic state, defined as an intermediate state between a localized tumor and widespread metastatic disease (12, 13). Landmark studies such as the SABR-COMET (7), have shown that adding SBRT to the standard of care improves overall survival (OS) by reducing the total cancer burden, debulking gross lesions sited near sensible structures and ultimately diminishing the number of neoplastic clones that need to be eradicated by systemic treatment. Another piece of evidence on the role of ablative radiotherapy comes from the STOMP trial, the first prospective randomized study assessing the potential of MDT, primarily SBRT, to forestall initiation of Androgen Deprivation Therapy (ADT) in hormone-sensitive oligometastatic prostate cancer patients presenting with three or fewer detectable metastases) (14). Similarly, a single-arm phase II trial of SBRT in oligometastatic disease demonstrated a 2-yr freedom from ADT of 48% (15). Moreover, the STAMPEDE trial showed that treating the primary tumor with radiotherapy improves OS, without detriment in QoL, in men with newly diagnosed low-burden metastatic prostate cancer, indicating that it should be recommended as a standard of care (16). Additional evidence regarding the importance of SBRT in low-burden disease comes from a *post-hoc* analysis of the KEYNOTE-001 study; in this trial, patients affected by non-small cell lung cancer (NSCLC) with metastatic disease showed improved OS if they received metastasis-directed therapy compared to the ones who didn’t (6 months OS was 73% vs. 45) (17). Based on available data from clinical trials, we currently therefore include SBRT as a valid treatment option which may be integrated with other treatment modalities in oligometastatic cancer patients. More specifically, the use of SBRT is based on the hypothesis that early ablation of metastatic disease may both prevent further seeding and influence metastatic crosstalk. Prostate cancer is a multifocal tumor with high intra-tumoral and inter-tumoral heterogeneity that can harbor or develop aggressive foci with time (18). Individual subclones can seed from the primary or from one metastasis to another in a polyclonal fashion, so that, every metastatic site can be considered a conglomerate of different subclones. In addition, it seems that metastasis-private mutations are associated with drug resistance (19). In this biological scenario, ablation of both the primary and oligometastatic tumor deposits can potentially eliminate sources for additional seeding events and could improve oncologic outcomes in patients with metastatic disease (20, 21). Regarding the tumor microenvironment, it has been hypothesized that SBRT induces an *in situ* vaccine response triggering the local activation of systemic antitumor immunity and promoting tumor-antigen expression and T-cell re-activation (22, 23). Considering the growing importance of cancer immunotherapy, numerous preclinical studies investigated the combination of a range of radiation regimens and immune checkpoint inhibitors. Lee et al. (24) explored the effect of high-dose single fractions which improves antigens presentation by antigen-presenting cells (APCs) and induces CD8+ T-cell priming. High doses of ionizing radiation induce pro-inflammatory signals via the cGAS-STING-IFN pathway, which could convert immune cells in the tumor microenvironments into phenotypes more amenable to T-cell trafficking but could also induce the release of a diverse array of neoantigens from the different metastatic sites into the blood circle (25–27). This hypothesis was reinforced by Diamond et al. findings which proved that even a lower dose of 8 Gy per fraction could also lead to antitumor T-cell responses in poorly immunogenic tumors, by promoting I IFN production and recruitment of dendritic cells into tumors (28).

Based on all these biological and clinical considerations, it may be plausible to expand this treatment paradigm in the setting of polymetastatic disease as well.

In the polymetastatic state, characterized by a widespread dissemination of metastases, patients are treated mainly with systemic therapies and may eventually become resistant to all different drug regimens. Recent advances in radiation oncology may give us the opportunity to raise the bar and allow the treatment of all sites of disease, overcoming the limit in terms of number of lesions that we imposed ourselves when we introduced the concept of oligometastases.

## Functional imaging: current status

A crucial issue of metastasis-directed therapy is that the imaging techniques used to detect the lesions should be accurate enough to define the actual metastatic burden. It is presumed that the more precise the disease extent definition is, the greater the percentage of patients receiving the appropriate MDT treatment would be, with expected improved oncological outcomes. In the 2017 Advanced Prostate Cancer Consensus Conference meeting (APCCC), oligometastatic prostate cancer was defined as the presence of three or fewer bone or lymph node metastases according to standard imaging modalities, including bone scintigraphy, contrast-enhanced computed tomography (CT) and morphological Magnetic Resonance Imaging (MRI) (29). However, although recommended by most guidelines, these techniques have poor diagnostic accuracy, underestimating the exact number of metastatic deposits (30). In this setting, functional imaging may represent an added value because it provides information on the biologically active cancer lesions and modern imaging techniques including PET/CT with specific tracers have demonstrated an improved detection rate (DR) for PCa recurrences compared to conventional imaging, especially for PSA levels less than 2 ng/mL (31).

Fluorine-18 (18F) or Carbon-11 (11C) radiolabeled choline was the first radiopharmaceutical agent employed in PCa evaluation. Choline is an essential nutrient involved in the synthesis of phosphatidylcholine, a vital component of the cell membrane. The increase in cell proliferation as well as the activity of the enzyme choline kinase in PCa cells is associated with an increase in choline uptake (32). For several years, it has been recommended by international guidelines as the gold-standard approach for PCa restaging in the presence of biochemical recurrence (BCR) (33). However, the clinical utility of choline-labeled PET/CT remains controversial in patients with early BCR. Treglia et al. in their meta-analysis, found that PSA doubling-time (PSAdt) ≤ 6 months and PSA levels >1 or >2 ng/mL/year proved to be relevant factors in predicting the positive result of radiolabeled choline PET/CT. The detection rate (DR) of radiolabeled choline PET/CT increased to 65% when PSAdt was ≤6 months and to 71% and 77% when PSA levels were >1 or >2 ng/mL/year, respectively (34). These data support the use of choline in this patient population, particularly for cases with PSA values exceeding 1 ng/mL.

The need for more accurate radiotracers led to the development of a new PET radiopharmaceutical and in May 2016, following its FDA approval, [18F]fluciclovine emerged as a useful molecular imaging agent in patients with PCa (35).

[18F]fluciclovine is a leucine analogue absorbed via the L-type amino acid transporter (LAT1) and the sodium-dependent neutral amino acid transporter (ASCT2), which is upregulated in many human cancers, including PCa (36).

When considering the impact of different biochemical parameters on prostate-radiopharmaceuticals DR, the [18F]fluciclovine PET/CT demonstrated superior performance in case of both low (0.5–1 ng/mL) and high PSA levels (>1 ng/mL). Additionally, PSAdt did not have significant impact on the effectiveness of [18F]fluciclovine DR, preserving a good performance also for PSAdt > 12 months (37).

However, [18F]fluorocholine PET/CT demonstrated better performance in the evaluation of the bone region (37).

In the last few years, the introduction of radiopharmaceuticals targeting Prostate-Specific Membrane Antigen (PSMA) has revolutionized diagnostic imaging of PCa (38). Positron-emitting radioisotopes such as Fluorine-18 (18F), Gallium-68 (68Ga), Copper-64 (64Cu), and Zirconium-89 (89Zr) all selectively bind to the extracellular domain of PSMA, which is typically overexpressed in PCa. 18F-based PSMA- targeted PET agents have the advantage of a longer radioisotope half-life (110 minutes), with increased positron yield, shorter positron range and good spatial resolution, favoring centralized radioisotope production and distribution (39, 40). However, there have not been large randomized clinical trials comparing these agents, and to date there is little clinical difference between them (41).

Also, the different radiochemistry may sometimes reflect a distinct physiologic and para-physiologic organ distribution. However, molecule leakage may also lead to non-specific uptake. Therefore, the PSMA ligand imaging analysis reports potential pitfalls (e.g. ganglia, benign bone lesions or non-specific lymph nodes). For example, 18F can leak out of the molecule leading to non-specific bone uptake but it also presents an advantage in distinguishing ureter or bladder radioactivity from local recurrence or locoregional metastases, due to minimally excreted concentration via the urinary tract in the case of 18F-PSMA-1007 (42, 43).

According to the recently published EANM/SNMMI guidelines (44), the radiopharmaceuticals 68 Ga-PSMA-11, 68 Ga-PSMA-I&T, 18F-DCFPyL, 18F-PSMA-1007, and 18F-rhPSMA-7.3 represent the most advanced imaging tools. Among them, 68 Ga-PSMA-11 (ILLUCCIX), 18F-DCFPyL (PYLARIFY/PYLCLARI), and 18F-rhPSMA-7.3 (POSLUMA) are already FDA and EU-approved.

Currently in the clinic, PSMA PET is predominantly indicated in recurrent or persistent prostate cancer, as a re-staging procedure following curative-intent therapy; it can also be employed as a primary staging procedure in high-risk disease and in the setting of castrate-resistant prostate cancer, which appears localized on conventional imaging. The factors associated with a higher DR are PSA, PSAdt, Gleason score and PSMA expression of the primary (45–49).

Concerning lymph nodes detection, PSMA PET has demonstrated a sensitivity and a specificity of 99% and 76%, respectively (50).

The value of the bone detection rate was also analyzed by several authors. Zhou et al. (51) obtained per-patient pooled sensitivity values of PSMA-PET/CT, choline-PET/CT, NaF-PET/CT, MRI, and BS; they were 0.97, 0.87, 0.96, 0.91, and 0.86, respectively, while the pooled specificities were 1.00, 0.99, 0.97, 0.96, and 0.95.

The key focus at present is to establish whether this improved diagnostic performance may guide changes in the therapeutic management of PCa patients. In their meta-analysis of 34 studies including 1057 patients with biochemical relapse-free survival, Pozdnyakov et al. demonstrated that PSMA PET can alter treatment plans in 56.4% of the population. BCR-free survival was 60.2% at median follow up of 20 months (52).

In another multicenter experience, PSMA PET showed a DR of 40.9% for PSA values of 0.2–0.4 ng/ml and 64.2% for values from 0.8 to 1 ng/ml (53).

Overall, these results make PSMA PET a very appealing tool for guiding MDT approach, especially in metachronous oligometastatic disease occurring after radical surgery; this therapeutic approach may not only provide local control of the treated lesions and potentially better oncological outcomes, but it can also ameliorate quality of life by delaying the onset of ADT and its related side effects (54, 55).

## Therapeutic options in mHSPC: general considerations

Metastatic hormone-sensitive prostate cancer (mHSPC) is an advanced stage of prostate cancer characterized by the presence of distant metastases and sensitivity to hormonal manipulation.

Although Androgen Deprivation Therapy (ADT) has historically represented the gold standard pillar for the treatment of this stage of disease, the therapeutic management of mHSPC has undergone significant changes especially during the last decade, leading to expanded treatment options for these patients and improved oncological outcomes (56). Concomitantly with the development of several new drugs, which have demonstrated the capacity of potentiating standard ADT and overcoming its initial mechanisms of resistance, a particular form of high intensity radiotherapy (SBRT) was shown to improve outcomes in the oligometastatic setting according to the results of specifically designed Phase II trials (14, 21) and one phase III trial (7).

## Systemic oncological therapy

Systemic oncological therapy plays a crucial role in the treatment of mHSPC. For several decades, androgen deprivation therapy (ADT) alone represented the standard approach (57), aiming to suppress the production or block the action of androgens, which fuel the growth of prostate cancer cells.

However, emerging evidence has demonstrated the benefits of combining ADT with other agents, such as chemotherapy and novel hormonal therapies (58). These systemic treatments target various pathways involved in prostate cancer growth and progression, offering the potential for enhanced tumor control and improved patient outcomes.

One of the trials that revolutionized the treatment landscape for mHSPC was the CHAARTED trial. This landmark study compared ADT alone versus ADT in combination with docetaxel, a taxane-based chemotherapy, in patients with newly diagnosed mHSPC (59). The trial demonstrated a significant improvement in overall survival and delayed disease progression in the combination arm, leading to the incorporation of docetaxel into the standard treatment regimen for mHSPC. Subsequent studies, including the STAMPEDE (60) and LATITUDE (61) trials, further supported the use of combination therapies by showing prolonged survival and delayed disease progression with the addition of abiraterone acetate, an androgen biosynthesis inhibitor, to ADT.

Moreover, the understanding of prostate cancer biology has led to the development of novel hormonal therapies targeting the androgen receptor pathway. Drugs such as enzalutamide and apalutamide, which act as androgen receptor antagonists, have demonstrated efficacy in improving survival outcomes and delaying disease progression in mHSPC (62, 63). These agents provide additional options for patients who may not be suitable for or have contraindications to chemotherapy.

Nonetheless, the landmark trial PEACE 1 (64) demonstrated that for selected patients presenting with *de novo*, high-volume, hormone-sensitive metastatic prostate cancer, triplet therapy with ADT, an androgen-receptor inhibitor, and docetaxel may represent the most efficacious strategy.

## Oligometastatic patients: the role of SBRT

The oligometastatic state refers to a stage of prostate cancer characterized by a limited number of metastases, typically confined to a few anatomic sites (6). In recent years, studies have explored the use of localized treatments, including ablative RT, to target and control these limited metastatic lesions (65). The rationale behind this approach is to eradicate or control oligometastatic disease, potentially delaying or preventing the need for systemic therapy, and improving long-term outcomes.

Concerning the role of SBRT, The COMET trial focused on evaluating the benefits of metastasis-directed therapy in a group of oligometastatic patients from different primitive tumors, including prostate cancer patients (7). The trial demonstrated a significant improvement in survival outcomes in patients receiving MDT. These findings have generated interest in the use of ablative RT as a potential therapeutic option for patients with oligometastatic prostate cancer, leading to ongoing research and exploration of the optimal timing, dose, and target selection for RT in this context.

Additionally, multiple retrospective studies and some phase II trials have reported encouraging outcomes with the use of SBRT in oligometastatic prostate cancer (66). These studies have shown that the addition of ablative radiotherapy to the oligometastases led to improved local control, progression free survival and delayed initiation of systemic therapy (14, 15). However, it is important to underline that further investigations in appropriate clinical trials will be crucial for determining optimal patient selection, radiation techniques, target volumes and dose fractionation schemes for these patients.

One of the arms of the STAMPEDE trial investigated the addition of RT to the primary tumor in patients with newly diagnosed mHSPC (16). Low metastatic burden was determined to be predictive of improved overall survival (OS) when radiation therapy (RT) was added to standard of care (SOC) therapy in these patients; of note, this benefit was not demonstrated in patients presenting with high burden metastatic disease. A subsequent subgroup analysis (67) revealed that the association of radiotherapy to the prostate + SOC did improve OS (p-value = 0.003) and failure-free survival (FFS) (treatment interaction p-value 0.001) in patients with only non-regional lymph nodes or < 4 bone metastases regardless of location and no visceral metastases over SOC alone.

## Biology guided radiotherapy: a new frontier in cancer therapy?

The RefleXion^®^ X1 biology-guided radiotherapy system (RefleXion Medical, Inc., Hayward, CA) combines dual imaging technologies (CT and PET) with a 6MV linear accelerator in a ring gantry (68). kVCT imaging is classically used for patient setup, while PET detects outgoing tumor emissions used to direct the therapy beam with sub-second latency. This interplay between the lesion and treatment machine allows the system to accurately guide and conform radiation beamlets to the tumor, taking into account its possible movement during treatment, to finally obtain a tracked dose distribution with the aim of sparing the irradiation of surrounding normal tissues. This has a profound impact on the classic radiotherapy workflow. Emissions from a tumor after radiotracer injection act as a fiducial, raising the confidence in target identification and reducing the need for positional margins to be added to the treated volumes during treatment planning. Besides, predictive motion models and motion management itself may be dramatically simplified and reduced by using a single radiotracer injection to manage motion during treatment to multiple sites of gross disease deposits throughout the body. The RefleXion biology-guided radiotherapy, referred to as *SCINTIX^®^ therapy*, has been FDA-cleared in 2023 for the treatment of lung and bone tumors (that could be primary or metastatic) using 18F-FDG. We can envision therapeutic avenues of this technology for metastatic prostate cancer patients, as well as the potential of using other radiotracers such as PSMA for SCINTIX therapy. Indeed, a preliminary study (69) reported promising results regarding the feasibility of [18F]-DCFPyL-guided BgRT planning. Twenty patients who were already scheduled for a PSMA diagnostic PET scan underwent a second scan on the RefleXion X1 to evaluate the quality of detection of [18F]-DCFPyL PSMA by the PET subsystem and the feasibility of [18F]-DCFPyL-guided BgRT planning. A PET avid tumor was identified and segmented for planning in 16 patients (4 lymph nodes, 5 bone, 6 prostate gland, and 1 prostate bed). Wong et al. demonstrated that BgRT planning was feasible and met standard of care stereotactic body radiotherapy (SBRT) organ dose constraints in 8 patients (3 prostate gland, 3 bone, 2 lymph nodes) (68).

In this context, Palma et al. recently designed a phase I trial analyzing the safety and tolerability of SBRT for the treatment of polymetastatic disease (70). If confirmed in future research, these results would therefore open new horizons for radiotherapy applications in the clinics, and BgRT may emerge as a valuable tool for the treatment of all metastatic sites with ablative doses in cases of polymetastatic cancer.

## What’s beyond BgRT in prostate cancer? future perspectives and conclusions

Notable technological advancements have enabled a prominent role for medical imaging in cancer treatments, specifically in radiation oncology (71). With the emergence of the field of radiomics, the interest of the scientific community in extracting quantitative image parameters for radiotherapy applications and prognostication is now increasing (72). In prostate cancer, several attempts have been made to find prognostic biomarkers at different time points of the disease, from diagnosis to metastases formation (**Table 1**). Of note, radiomic features extracted from PET images may provide valuable information for better patient selection and response to therapies. Furthermore, integrating functional imaging in the BgRT workflow may pave the way towards potentially relevant clinical applications in the field of radiotherapy, ultimately leading to a more personalized approach and plan optimization. The Dominant Intraprostatic Lesion (DIL) visible at functional imaging and/or MRI in localized prostate cancer, represents an example of when a biology-guided prescription and delivery of a non-uniform dose to the clinical target volume (CTV) is beneficial; boosting the intraprostatic lesion was shown to improve biochemical disease-free survival in patients with localized prostate cancer without impacting toxicity and quality of life (88). Likewise, the functional information obtained with molecular imaging in metastatic prostate cancer may not only reduce inter-observer variability in target volume delineation and decrease radiation therapy planning time, but it can also guide dose escalation to potential tumor sub volumes with increased radioresistance or higher tumor burden. Gaudreault et al. (89) have explored the possibility of integrating LuPSMA therapy with BgRT in metastatic CRPC patients who have PSMA-negative/FDG-positive PET imaging; more specifically, these patients are not likely to benefit from radionuclide therapy because some disease sites may not represent a target due to inadequate PSMA uptake, but they may be good candidates for FDG PET-guided BgRT.

**Table 1:** Selected MRI and PET-based radiomic studies in PCa.

Author, Year	Study Phase	N° patients	Imaging	Patient Cohort	Outcome Measures	External Validation	Results Synopsis
Liu, 2022 (73)	R	537	MRI (T2w, DWI)	PCa with presence of pelvic lymph node M	Pelvic Lymph Node Metastases		Model 2 showed the highest AUC =0.83 ( 95% CI, 0.76, 0.89)
Mattoni, 2022 (74)	R	60	68Ga-PSMA PET	mCRPC M+ (liver)	Liver M detection	No	AUC = 0.807 (95% CI, 0.686–0.920); spec = 0.87; sens = 0.75
Kairemo, 2022 (75)	R	14	18F-NaF-PET/68Ga-PSMA-11-PET	PCa M+ (bone)	Bone M detection	No	NaFand PSMA suit the evaluation of active skeletal disease and actually provide complementary information
Assadi, 2022 (76)	R	33	68Ga-PSMA PET	mCRPC patients undergoing 177Lu-PSMA therapy	BCR, OS	No	BCR GLCM entropy (cut-off 7.405; sens 82%; spec 73%) (AUC= 0.719); Age (AUC = 0.749); treatment cycle (AUC = 0.838); administered dose (AUC = 0.827)
Hou, 2021 (77)	R	401	MRI (T2w, DWI, ADC)	PCa patients who underwent RP & ePLND	Pelvic Lymph Node Metastases	Yes	AUC = 0.76 (95% CI, 0.62-0.87)
Alongi, 2021 (78)	R	94	18F-Choline PET	High Risk PCa	LNI, Distant Metastasis	No	LNI AUC = 69.87 (95% CI 51.34 - 88.39) Distant Metastasis AUC = 74.72 (95% CI 56.36 - 93.09)
Moazemi, 2021 (79)	R	83	68Ga-PSMA PET/CT	Advanced PCa undergoing 177Lu-PSMA therapy.	OS	No	PET Kurtosis & SUVmin significantly correlated with OS
Zhang,2020 (80)	R	116	MRI (T2w, DCE, T1w, DWI)	Biopsy confirmed PCa	Bone Metastases	No	AUC = 0.93 (95% CI, 0.86 – 0.99)
Cysouw, 2020 (81)	P	76	18F-DCFPyL PET	Intermediate/HR PCa	LNI, Distant Metastasis	No	LNI AUC = 0.86 ± 0.15, p < 0.01 Distant Metastasis AUC = 0.86 ± 0.14, p < 0.01
Wang, 2019 (82)	R	176	MRI (T2w, DCE, T1w)	PCa M-	Prediction of Bone Metastasis	No	AUC = 0.895 (95% CI 0.836 - 0.939)
Lin, 2019 (83)	P	14	18F-NaF PET	mCRPC patients under ADT	R/R	No	Skewness, Kurtosis and diagonal moment exhibited greater R/R than SUVmax
Zhao, 2019 (84)	R	193	68Ga-PSMA PET/CT	mCRPC	Pelvic M detection	No	Precision range 0.79 – 0.99 depending on the type of lesion detect (bone, lymph node, or local) Sensitivity range 0.61 – 0.99
Reischauer, 2018 (85)	P	12	MRI (ADC)	Treatment-naïve advanced PCa with scintigram M+	Change in response to ADT therapy	No	1st- and 2nd-order statistical features showed promise
Khurshid, 2018 (86)	R	70	68Ga-PSMA PET	mCRPC patients planned to undergo 177Lu-PSMA therapy	Correlation with treatment response	No	Entropy and Homogeneity correlated with response (r = -0.327 & r = 0.315, respectively)
Perk, 2018 (87)	R	37	18F-NaF PET/CT	mCRPC patients	Bone Lesion Classification	No	AUC = 0.95 (95% CI 0.93 – 0.96)

Acc, Accuracy; AUC, Area Under Curve; CSS, Cause-specific survival; HR, Hazard Ratio; NS, Not Specified; OS, Overall Survival; R/R, Response-to-repeatability; Sens, Sensitivity; Retrospective, R; Prospective, P; Spec, Specificity; ADT, Androgen deprivation therapy; ePLND, extended pelvic lymph node dissection; NPV, Negative Predictive Value; PPV, Positive Predictive Value; RP, radical prostatectomy; ePLND, extended pelvic lymph-node dissection; M, metastasis; mCRPC, metastatic castration resistant prostate cancer.

In conclusion, we believe that BgRT, already a new addition in the armamentarium against lung and bone tumors using 18F-FDG, could represent a potentially valuable add-on in the integrative therapy management of prostate cancer patients using a PSMA radiotracer. This is particularly relevant in the setting of metastatic disease, where we are already exploring offering metastasis directed therapy in a polymetastatic state (70).

Further research is warranted to confirm the safety and the efficacy of this fascinating therapeutic hypothesis.
